# Innate immune responses and neuroepithelial degeneration and regeneration in the mouse olfactory mucosa induced by intranasal administration of Poly(I:C)

**DOI:** 10.1007/s00441-014-1848-2

**Published:** 2014-04-18

**Authors:** Kaori Kanaya, Kenji Kondo, Keigo Suzukawa, Takashi Sakamoto, Shu Kikuta, Kazunari Okada, Tatsuya Yamasoba

**Affiliations:** Department of Otolaryngology-Head and Neck Surgery, The University of Tokyo Graduate School of Medicine, 7-3-1 Hongo, Bunkyo-ku, Tokyo 113-8655 Japan

**Keywords:** Postviral olfactory disorder, Toll-like receptor 3, Neutrophil, Elastase, Immunohistochemistry

## Abstract

The pathogenesis of postviral olfactory disorder (PVOD) has not been fully elucidated. We investigated morphological changes and innate immune responses in the mouse olfactory mucosa induced by intranasal administration of polyinosinic-polycytidylic acid [Poly(I:C)], a synthetic analog of viral double-stranded RNA. Mice received three administrations of saline with or without Poly(I:C), once every 24 h. The olfactory mucosa was harvested at various intervals after the first administration (8 h, 3, 9 and 24 days). In the Poly(I:C) group, the number of apoptotic cells in the olfactory neuroepithelium had increased at 8 h. At 9 days, the olfactory neuroepithelium had severely degenerated and behavioral tests demonstrated that the mice showed signs of olfactory deterioration. At 24 days, the structure of the neuroepithelium had regenerated almost completely. Regarding the innate immune responses, many neutrophils had infiltrated the olfactory neuroepithelium at 8 h and had exuded into the nasal cavity by 3 days. Macrophages had also infiltrated the olfactory neuroepithelium at 8 h although to a lesser extent, but they still remained in the neuroepithelium at 24 days. Poly(I:C)-induced neuroepithelial damage was significantly inhibited by a neutrophil elastase inhibitor and was suppressed in neutropenic model mice. These findings suggest that the secondary damage caused by the neutrophil-mediated innate immune response plays an important role in the pathogenesis of PVOD.

## Introduction

The olfactory mucosa has a unique feature: it is a nervous tissue as well as a part of the airway and is constantly exposed to various pathogens. The olfactory mucosa generates new olfactory receptor neurons (ORNs) through normal turnover, and also after injury, in order to maintain its function (Graziadei and Graziadei 1979; Schwob 2002). The ORNs are produced by proliferation of globose basal cells with newly generated neuronal cells migrating toward the surface of the olfactory neuroepithelium during maturation. The ORNs express specific molecular markers depending on their maturational status. Immature ORNs express growth-associated protein (GAP)-43 or neuron-specific βIII tubulin (βIIIT) (Verhaagen et al. 1990; Lee and Pixley 1994; Roskams et al. 1998), whereas mature ORNs express olfactory marker protein (OMP) (Monti Graziadei 1983; Kream and Margolis 1984).

In spite of such a regenerative capacity, a number of pathological conditions can cause olfactory dysfunction in humans. Postviral olfactory disorder (PVOD) is defined as an olfactory dysfunction following a viral upper respiratory tract infection (URI) and is one of the most common causes of olfactory dysfunction (Mott and Leopold 1991; Collet et al. 2009). PVOD is considered to be caused by damage to the nervous tissues of the olfactory pathways, which may not have fully recovered from the initial functional damage. It can also cause unpleasant olfactory symptoms such as parosmia and phantosmia (Doty 1979).

Although the precise molecular mechanisms of tissue damage in PVOD have not been fully elucidated, two mechanisms have been postulated. One is direct virus-induced damage to the olfactory neuroepithelium or olfactory central pathways (Seiden 2004); the other is secondary damage due to host immune responses. Most of the studies regarding PVOD have focused on virus-induced apoptosis of the olfactory neuroepithelium or the olfactory bulb (Schwob et al. 2001; Welge-Lussen and Wolfensberger 2006), while there have been few studies focusing on the damage caused by immune responses.

In viral infections, host cells recognize viral components via Toll-like receptors (TLRs), a family of innate immune receptors that recognize pathogen-associated molecular patterns (Akira 2001). Various viruses produce double-stranded (ds) RNAs during their replication cycle (Jacobs and Langland 1996), which are recognized by TLR3. The stimulation of TLR3 causes the translocation of interferon regulatory factor-3 (IRF-3) and nuclear factor (NF)-κB into the nucleus and the subsequent release of type I interferon and proinflammatory cytokines including TNFα, IL-6 and IL-8 (Alexopoulou et al. 2001; Matsukura et al. 2006). These cytokines induce migration and activation of inflammatory cells. In general, immune system processes work to protect the host against pathogens, but excessive immune response might itself cause tissue damage. Primed neutrophils release various inflammatory mediators, such as neutrophil elastase (NE) and cathepsin G, into extracellular spaces, and NE in particular is thought to be mainly involved in tissue damage (Janoff 1985; Inoue et al. 2006).

To address the secondary damage by host immune responses in PVOD, we investigated morphological changes of the olfactory mucosa and innate immune responses induced by intranasal administration of a synthetic dsRNA, polyinosinic-polycytidylic acid [Poly(I:C)], which has been established as an immune model of viral infection (Jacobs and Langland 1996; Matsumoto and Seya 2008). To elucidate the contribution of neutrophils to tissue damage, we also performed three experiments to determine: (1) how the olfactory neuroepithelium could be injured by the intranasal administration of elastase itself, (2) whether Poly(I:C)-induced neuroepithelial damage could be suppressed by a neutrophil elastase inhibitor, and (3) whether Poly(I:C)-induced neuroepithelial damage could be suppressed in a neutropenic murine model treated with cyclophosphamide, which induces myelosuppression.

## Materials and methods

### Animals

Female ICR mice at postnatal age of 3 months, obtained from Saitama Experimental Animals (Saitama, Japan), were used. We chose female mice because postviral olfactory disorder in humans occurs predominantly in women (Sugiura et al. 1998). They were housed in a temperature-controlled environment under a 12 h light–dark cycle with free access to food and water. All procedures were approved by the University of Tokyo Animal Care and Use Committee, and performed in accordance with the National Institute of Health Guide for the Care and Use of Laboratory Animals.

### Intranasal administration of Poly(I:C)

**Fig. 1:**
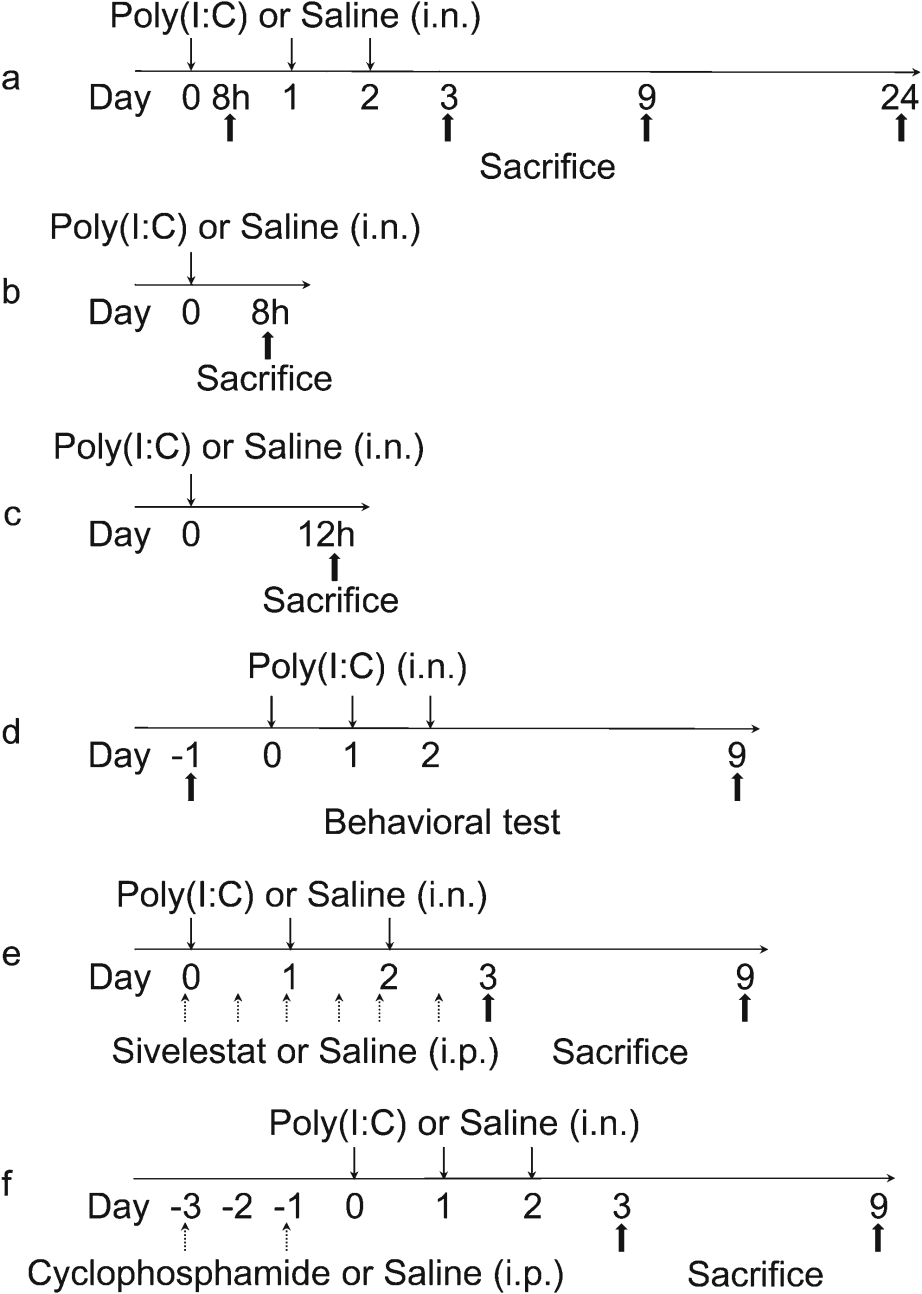
Experimental protocols. **a** Protocol for intranasal administration of Poly(I:C) or saline. Mice received three intranasal administrations of Poly(I:C) or saline, once every 24 h, and were sacrificed at 8 h, 3, 9 and 24 days after the first administration (*n* = 3/group). **b** Protocol for examination of phospho-IRF-3(Ser 396) expression by western blot.The olfactory mucosae of the ethmoturbinates were harvested 8 h after single intranasal administration of Poly(I:C) or saline.** c** Protocol for measurement of MIP-2 levels by ELISA. The olfactory mucosae of the ethmoturbinates were harvested 12 h after single intranasal administration of Poly(I:C) or saline (*n* = 4/group). **d** Protocol for behavioral testing. We performed an olfactory habituation/dishabituation test before and 9 days after the intranasal administration of Poly(I:C) in the same mice (n = 5). **e** Protocol for administration of Poly(I:C) and Sivelestat. Mice received intraperitoneal administration of Sivelestat or saline 30 min before and 12 h after every intranasal administration of Poly(I:C) or saline and were sacrificed at 3 and 9 days after the first administration of Poly(I:C) (*n* = 3/group). **f** Protocol for administration of cyclophosphamide and Poly(I:C). Mice received intraperitoneal injections of cyclophosphamide or saline twice every other day. After the final injection, mice were administered Poly(I:C) or saline intranasally three times every 24 h and were sacrificed at 3 and 9 days after the first administration (*n* = 3/group)

Mice were anesthetized with an intramuscular combined injection of ketamine (80 mg/kg) and xylazine (9 mg/kg) before receiving intranasal administration of 50 μg Poly(I:C) sodium salt (P1530; Sigma-Aldrich, Japan) dissolved in 25 μl of sterile saline into the left naris (this experimental group was designated as the Poly(I:C) group). The dose of Poly(I:C) used was determined from the doses used in previous studies of respiratory mucosa (Stowell et al. 2009), since there were no previous data about the effect of Poly(I:C) on olfactory mucosa. The control mice received saline alone in the same manner (this experimental group was designated as the control group). Each mouse received three administrations of saline with or without Poly(I:C), once every 24 h, and were sacrificed as described below at various intervals after the first administration (8 h, 3, 9 and 24 days; *n* = 3/group). The experimental protocol is shown in Fig. 1a.

### Fixation and tissue preparation

Mice were deeply anesthetized using a combination of ketamine and xylazine, fixed by cardiac perfusion with 10 % neutral buffered formalin (Muto Kagaku, Tokyo, Japan), and then decapitated. Their nasal cavities were locally irrigated with the same fixative by means of a 1 ml syringe with a 26G nonbevel needle. The lower jaws were discarded, after which the trimmed heads were skinned and further fixed by immersion in the same fixative for 1 week at room temperature (RT). Then, the specimens were decalcified in 10 % ethylenediamine tetraacetic acid (EDTA, pH 7.0) for 2 weeks at RT, washed, dehydrated in a graded ethanol series, and embedded in paraffin. Serial coronal sections (4 μm thick) were cut and mounted on MAS-coated slides (Matsunami Glass, Osaka, Japan). These sections were stained with hematoxylin and eosin (H&E), or immunohistochemically as described below.

### Primary antibodies

A list of all primary antibodies used is shown in Table 1. Rabbit polyclonal anti-TLR3 antibody obtained from Abcam (#ab53424; Cambridge, UK) was developed using a mix of synthetic peptides corresponding to amino acids 135–150 (SIHKIKSNPFKNQKNL), 828–844 (CRRFKVHHAVQQAIEQN), and 876–891 (CILNWPVQKERINAFH) of mouse TLR3. This antibody recognizes a band of approximately 104 kDa on western blots of mouse spleen tissue lysate (manufacturer’s technical information).

**Table 1:** Primary Antibodies Used

Antigen	Immunogen	Manufacturer	Dilution
Toll-like receptor 3	A mix of synthetic peptides correponding to amino acids 135–150 (SIHKIKSNPFKNQKNL), 828–844 (CRRFKVHHAVQQAIEQN), and 876–891 (CILNWPVQKERINAFH) of mouse TLR3	Abcam (Cambridge, UK), rabbit polyclonal, #ab53424	1:200
phospho-NF-_K_B p65 (Ser 276)	A synthetic phosphopeptide corresponding to residues surrounding Ser 276 of human NF-_K_B p65	Cell Signaling Technology (Danvers, MA, USA), rabbit polyclonial, #3037	1:400
Cleaved caspase-3	A synthetic KLH-coupled peptide (CRGTELDCGIETD) adjacent to Asp 175 in human caspase-3	Cell Signaling Technology (Danvers), rabbit polyclonial, #9661	1:400
Olfactory marker protein (OMP)	Purified rodent OMP	Wako chemical USA (Richmond, VA, USA), goat polyclonal, #544-10001	1:8,000
Beta III tubulin (βIIIT)	A synthetic pepetide corresponding to amino acids 443–450 (ESESQGPK) of human class III beta-tubulin	Millipore (Billerica, MA, USA), mouse monoclonal, #MAB1637	1:400
Ki67	22-amino-acid ki67 repeat motif (APKEKAQPLEDLASFQELSQ)	BD Bioscience (San Jose, CA, USA), mouse monoclonal (Clone B56), #550609	1:400
Neutrophil	Highly purified BALB/c mouse neutrophils	Abcam (Cambridge, UK), rat monoclonal (Clone NIMP-R14), #ab2557	1:400
F4/80	Mouse thioglycollate-stimulated peritoneal macrophages	AbD Serotec (Oxford, UK), rat monoclonal (Clone CI:A3-1), #MCA497	1:100
CD3	A synthetic peptide spanning amino acids 156–168 of the cytoplasmic domain of human CD3ε chain	Nichirei Corporation (Tokyo, Japan), rabbit monoclonal (Clone SP7), #413601	1:400
Phospho-IRF-3 (Ser 396)	A Synthetic phosphopeptide corresponding to residues surrounding Ser 396 of human IRF-3	Cell Signaling Technology (Danvers), rabbit monoclonal, #4947	1:2,000
β-actin	A KLH conjugated synthetic peptide corresponding to N-terminus of β-actin	MBL (Nagoya, Japan), rabbit polyclonal, #PM053	1:10,000

Rabbit polyclonal anti-phospho-NF-κB p65 (Ser 276) antibody obtained from Cell Signaling Technology was produced by immunizing animals with a synthetic phosphopeptide corresponding to residues surrounding Ser 276 of human NF-κB p65. This antibody recognizes a band of approximately 80 kD on western blots of extracts from NIH/3 T3 cells.

Rabbit polyclonal anti-cleaved caspase-3 antibody obtained from Cell Signaling Technology was raised against a synthetic KLH-coupled peptide (CRGTELDCGIETD) adjacent to Asp175 in human caspase-3, and is a well-defined marker of apoptosis in mammalian tissues (Ribera et al. 2002). The antibody recognizes 17- to 19-kD fragments, but not the full-length caspase-3 on western blots of human and mouse cell line homogenates (manufacturer’s data sheet).

The OMP antiserum (544–10001, Wako Chemical, Richmond, VA, USA) is a goat polyclonal antibody that was produced by immunizing animals with purified rodent OMP (Keller and Margolis 1975). It recognizes a single band of 19 kD molecular weight corresponding to OMP on western blots of mouse and rat olfactory bulb (Baker et al. 1989). It immunolabels a cell population that is morphologically classified as mature olfactory neurons in the rodent olfactory neuroepithelium (Verhaagen et al. 1989; Schwob et al. 1992; Kondo et al. 2009).

The βIIIT antiserum (MAB1637; Millipore, Billerica, MA, USA) is a mouse monoclonal antibody raised using a synthetic peptide corresponding to amino acids 443–450 (ESESQGPK) of human class III beta-tubulin as an immunogen. It recognizes a single band of 55 kD molecular weight corresponding to βIIIT on western blots of mouse brain lysate (manufacturer’s technical information). It recognizes immature ORNs and its staining pattern is similar to that of growth-associated protein-43 distribution (Lee and Pixley 1994; Roskams et al. 1998).

Mouse monoclonal anti-Ki67 antibody obtained from BD Biosciences (clone B56, #550609; San Jose, CA, USA) was developed using a highly conserved 22 amino acid element called the “Ki67 motif” of the human Ki67 protein. This antibody detects a double band of 345 and 395 kD on western blot analyses of human cells, consistent with the molecular weights of alternatively spliced Ki67 isoforms (Schluter et al. 1993). This antibody recognizes a cell proliferation-associated nuclear antigen expressed in all active stages of the cell cycle and immunohistochemical analysis demonstrates that B56 gives the same staining pattern as MIB1 on both frozen and paraffin-embedded tissue sections (manufacturer’s technical information).

Rat monoclonal anti-neutrophil antibody obtained from Abcam (clone NIMP-R14, #ab2557; Cambridge, UK) was produced by using highly purified BALB/c mouse neutrophils as an immunogen. The antibody recognizes full length of the surface protein Ly-6G.

Rat monoclonal anti-F4/80 antibody obtained from AbD Serotec (clone Cl:A3-1, #MCA497; Oxford, UK) was produced using mouse thioglycollate-stimulated peritoneal macrophages as an immunogen. This monoclonal antibody precipitates a 160-kD cell surface glycoprotein (Austyn and Gordon 1981) corresponding to the murine F4/80 antigen and labels macrophages in tissues of wild-type but not F4/80−/− mice (Lin et al. 2005).

Rabbit monoclonal anti-CD3 antibody obtained from Nichirei Corporation (clone SP7; Tokyo, Japan) was raised against a synthetic peptide spanning amino acids 156–168 of the cytoplasmic domain of human CD3ε chain. The CD3 antigen is present on early thymocytes and mature T cells and is generally regarded as a Pan T cell marker.

Rabbit polyclonal anti-phospho-IRF-3 (Ser 396) antibody obtained from Cell Signaling Technology was produced by immunizing animals with a synthetic phosphopeptide corresponding to residues surrounding Ser 396 of human IRF-3. This antibody recognizes a single band of 44–55 kD on western blots of extracts from HT29 and THP1 cells (manufacturer’s data sheet).

Rabbit polyclonal anti-β-actin antibody obtained from Medical & Biological Laboratories (#PM053; Nagoya, Japan) was raised against a KLH conjugated synthetic peptide corresponding to the N-terminus of β-actin. This antibody recognizes a single band of 42 kD on western blots of HeLa cell lysates (manufacturer’s data sheet).

### Immunohistochemistry

Sections were deparaffinized then rehydrated through a xylene and ethanol series. Sections for TLR3, cleaved caspase-3, OMP, Ki67, phospho-NF-κB p65 and neutrophil immunostaining were immersed in 10 mM citrate buffer solution (pH 6.0; Dako Cytomation, Kyoto, Japan) and autoclaved at 121°C for 10 min for antigen retrieval. Sections for F4/80 and βIIIT immunostaining were immersed in Antigen Retrieval Solution (S1700; Dako Cytomation) and sections for CD3 immunostaining were immersed in Antigen Retrieval Solution High pH (S3308; Dako Cytomation), and were then autoclaved in the same manner.

Endogenous peroxidase activity was blocked by treatment with 3 % hydrogen peroxide in methanol for 10 min at RT. All sections except those for βIIIT and Ki67 immunostaining were incubated for 10 min with blocking solution [Tris-buffered saline (TBS), pH 7.4, containing 2 % bovine serum albumin (Sigma-Aldrich Japan), 0.1 % Triton X-100 and 0.1 % sodium azide] at RT to reduce nonspecific antibody binding. The sections were then incubated with each primary antibody at 4°C overnight. After being washed in phosphate-buffered saline (PBS; pH 7.4), the sections were incubated for 1 hour at RT with horseradish peroxidase (HRP)-conjugated anti-rabbit IgG, anti-goat IgG or anti-rat IgG secondary antibodies (Simplestain MAX-PO [R], [G] [Rat], ready-to-use; Nichirei, Tokyo Japan), corresponding to the primary antibodies. In the βIIIT and Ki67 immunostaining procedure, an HRP-conjugated secondary antibody kit for immunostaining of mouse tissue with mouse primary antibodies (Simplestain mouse stain kit; Nichirei) was used according to the instructions of the manufacturer in order to prevent non-specific binding of the secondary antibody to endogenous mouse immunoglobulins.

After several washes in PBS (pH 7.4), immunoreactivity was made visible by the diaminobenzidine (DAB) reaction (Simplestain DAB, ready-to-use; Nichirei). After being washed with distilled water, sections were counterstained with hematoxylin, then dehydrated, and mounted. There was no obvious immunoreactivity when the primary antibodies were omitted from the staining procedure (data not shown).

For double-immunofluorescence staining of cleaved caspase-3 and OMP, sections were incubated with a mixture of primary antibodies at 4°C overnight, washed in PBS and incubated for 1 hour at RT with a mixture of secondary antibodies directed to each primary antibody used: Alexa Fluor 488-conjugated donkey anti-goat IgG (H+L) (1:100; Invitrogen) and Alexa Fluor 594-conjugated donkey anti-rabbit IgG (H+L) (1:100; Invitrogen). After several rinses in PBS, sections were coverslipped using Vectashield mounting medium (Vector Laboratories).

### Alcian blue staining

To investigate mucus secretion from Bowman’s glands induced by Poly(I:C), Alcian blue staining was performed on the sections of the Poly(I:C) group and the control group. Sections were deparaffinized, rehydrated and incubated in 3 % acetic acid solution for 3 min. The sections were then incubated with Alcian blue solution (pH 2.5; Muto Chemicals, Tokyo, Japan) for 2 h at RT. After several washes, sections were counterstained with nuclear fast red solution, then dehydrated, and mounted.

### Western blot analysis of phospho-IRF-3 expression

The olfactory mucosae of the ethmoturbinates were harvested 8 h after intranasal administration of saline or Poly(I:C) and were homogenized with 10 times as much volume of CelLytic ™ MT Cell Lysis Reagent (Sigma-Aldrich Japan) with a protease inhibitor cocktail (P8340; Sigma-Aldrich Japan) and benzonase endonuclease (E1014; Sigma-Aldrich Japan) (*n* = 3/group). Homogenized samples were centrifuged at 4°C at 15,000*g* for 10 min and the supernatants were obtained. Samples were mixed with sample buffer (TEFCO, Tokyo, Japan) and heated for 5 min at 95°C. Equal amounts of total protein were loaded into each lane and separated by 10 % sodium dodecyl sulfate-polyacrylamide gel electrophoresis (SDS-PAGE) and then transferred to a polyvinylidene difluoride (PVDF) membrane. The membrane was blocked with 0.3 % skim milk in 25 mM Tris–HCl buffer, pH 7.6, containing 0.15 M NaCl and 1 % Tween 20 (TBST) for 1 h at RT with gentle shaking. It was incubated with the following primary antibodies: rabbit anti-phospho-IRF-3 (Ser 396) antibody (1:2,000) and rabbit anti-β-actin antibody as a loading control (1:10,000) at 4°C overnight. The membranes were then washed and incubated with horseradish peroxidase (HRP)-conjugated goat anti-rabbit IgG antibody (1:10,000; Amersham Pharmacia Biotech) for 1 h at RT. Immunoreactivity was detected using an enhanced chemiluminescence (ECL) kit (Amersham Pharmacia Biotech) and captured using a LumiCube chemiluminescence analyzer (Liponics, Tokyo, Japan). Phospho-IRF-3 band densities were quantified using JustTLC image analysis software (Liponics) and normalized to β-actin. This protocol is shown in Fig. 1b.

### ELISA for Macrophage inflammatory protein-2 (MIP-2)

The olfactory mucosae were harvested 12 h after intranasal administration of saline or Poly(I:C) and were homogenized in the same manner as in the western blot analysis described above. After centrifugation, supernatants were obtained and MIP-2 (mouse homolog of IL-8) concentrations were determined with an enzyme-linked immunosorbent assay (ELISA) kit (Biosensis) according to the manufacturer’s instructions (*n* = 4/group). Absorbance was read at 450 nm on a microplate reader. This protocol is shown in Fig. 1c.

### Behavioral testing to evaluate olfactory function

To evaluate olfactory sensitivity, we performed an olfactory habituation/dishabituation test (Kobayakawa et al. 2007) with some minor modifications. Each mouse was tested before and 9 days after the intranasal administration of Poly(I:C) (*n* = 5). This protocol is shown in Fig. 1d. We selected the 9th post-treatment day because the number of mature olfactory receptor neurons was the lowest at this time point. This behavioral test relies on the mouse’s natural tendency to investigate novel smells. Briefly, mice were acclimated to a clean plastic cage (46 cm × 23.5 cm × 20 cm) without wood chip bedding for 30 min, and then presented with a filter paper soaked in mineral oil placed in a 35 mm polystyrene dish for 3 min. This procedure was repeated three times at 1 min intervals. On the fourth trial, a filter paper soaked instead with propyl propionate was presented for 3 min. The mouse behavior was recorded with a digital video camera and each investigation time was measured. Investigation was defined as the mouse’s nose being within 1 mm of the filter paper. The investigation time on the fourth trial was compared to that on the third trial. Mice with normal olfaction show significantly reduced sniff times when an odor is reintroduced for the second and third time (habituation), but they show a reinstatement of sniffing when a novel odor is presented. A lack of this reinstatement indicates the decrease or absence of olfactory sensitivity.

### Intranasal administration of elastase

Mice were anesthetized and then received an intranasal administration of 25 μl saline solution containing 0.1 mg elastase (type I elastase from porcine pancreas; Sigma-Aldrich Japan) three times every 24 h. The mice were sacrificed at 3 and 9 days after the first administration and their nasal tissues were harvested and processed for paraffin sectioning as described above (*n* = 3/group).

### Inhibition of neutrophil elastase

Sivelestat {ONO-5046; sodium N-[2-(4-[2, 2-dimethylpropionyloxy] phenylsulfonylamino) benzoyl] aminoacetate tetrahydrate; Ono Pharmaceuticals, Osaka, Japan} is a low molecular weight synthetic inhibitor of neutrophil elastase. Sivelestat (10 mg/kgBW) or saline was administered intraperitoneally 30 min before and 12 h after every third administration of Poly(I:C) or saline (these experimental groups were designated as the Sivelestat-Poly(I:C) group, the Saline-Poly(I:C) group or the Sivelestat-Saline group). Mice were sacrificed at 3 and 9 days after the first administration (*n* = 3/group) and were processed for paraffin sectioning. We then performed immunohistochemistry on the sections to compare the number of neutrophils and the degree of damage to the olfactory neuroepithelium among the three groups. This protocol is shown in Fig. 1e.

### Intranasal administration of Poly(I:C) to a neutropenic murine model

Mice were injected intraperitoneally with cyclophosphamide (CPA; 200 mg/kgBW; Shionogi, Osaka, Japan) or saline twice, with a 1 day interval (Oishi et al. 1992). The day after the last injection, we confirmed that leukocyte counts in the peripheral blood of CPA-injected mice were very low and then administered Poly(I:C) or saline intranasally three times, once every 24 h (these experimental groups were designated as the CPA-Poly(I:C) group, the CPA-Saline group or the Saline-Poly(I:C) group). The mice were sacrificed at 3 and 9 days after the first administration and examined for the infiltration of neutrophils and damage to the olfactory neuroepithelium (*n*= 3/group). This protocol is shown as Fig. 1f.

### Image presentation

Preparations of stained sections were examined using a microscope (E800; Nikon, Tokyo, Japan) under bright-field illumination or fluorescent illumination using green (excitation wavelengths: 510–560 nm; emission wavelength: 515–555 nm) and red (excitation wavelengths: 465–495 nm; emission wavelength: 590 nm) filters and photographed using a digital microscope camera (AxioCam; Carl Zeiss, Tokyo, Japan). Digital images were processed in Adobe Photoshop (Tokyo, Japan), adjusting only brightness, contrast, and color balance.

### Quantitative analysis

To control the variability, the quantitative analyses described below were made on coronal sections through the anterior end of the olfactory bulb. The number of cleaved caspase 3-, OMP-, Ki67-, and βIIIT-positive cells in the olfactory mucosa was manually counted in three high-magnification microscopic fields (×400) along the II, IV, and V ethmoturbinates of the left nasal cavity by a blinded observer and the average number of positive cells across the three fields was used for comparisons. We selected these regions for analyses because they were observed to be the most severely injured in the sections examined (see “Results”). The number of neutrophils, macrophages, and T lymphocytes was counted along the entire length of the left olfactory mucosa and in the left nasal cavity. To compare the amount of mucus in the Bowman’s glands, we selected three random high-magnification microscopic fields (×400) along the II, IV, and V ethmoturbinates and measured the cross-section area of Alcian blue-positive mucus in the Bowman’s glands using image analysis software (ImageJ, NIH) (*n* = 3/group). The area was expressed as μm^2^ per mm of neuroepithelial length and the total area from the three fields was compared between the Poly(I:C) group and the control group.

### Statistical analysis

Each result was described as a mean ± SEM of the samples. Data were statistically evaluated using SPSS statistical software (SPSS, Chicago, IL, USA). The difference in the number of OMP- or Ki67-positive cells among the groups in the Sivelestat experiment and the groups in the cyclophosphamide experiment was compared using a one-way analysis of variance (ANOVA) followed by the Bonferroni *t* test. The difference in MIP-2 levels or phospho-IRF-3 expression was compared using the Student’s *t* test. The difference in the investigation time between the third and fourth trials in the olfactory habituation/dishabituation test was evaluated using a paired *t* test. The difference in the area of Alcian blue staining between the Poly(I:C) group and the control group was evaluated using the Student’s *t* test. A *p* value  < 0.05 was considered statistically significant.

## Results

### Expression of TLR3 and its downstream signals in the mouse olfactory mucosa

**Fig. 2:**
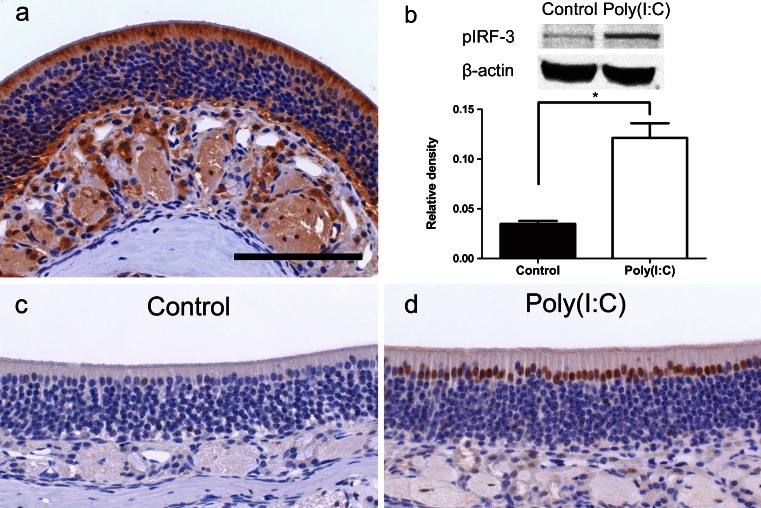
Expression of TLR3, phospho-IRF-3 (Ser 396) and phospho-NF-κBp65 (Ser 276) in the mouse olfactory mucosa. **a** The expression of TLR3 was observed mainly in the apical part of the supporting cells and in the cytoplasm of the acinar cells of Bowman’s glands.** b** Western blot image showing phospho-IRF-3 expression. *Bar graph* represents the relative density of each band normalized to β-actin as internal control. It was significantly greater in the Poly(I:C) group than in the control group (*n* = 3, *p* < 0.005). **c** In the control group, immunolabeling for phospho-NF-κBp65 was observed very weakly in the nuclei of some supporting cells. **d** In the Poly(I:C) group, immunolabeling for phospho-NF-κBp65 was detected intensely in the nuclei of many supporting cells and acinar cells of Bowman’s glands at 8 h. *Bar* 150 μm

The expression of TLR3 in the olfactory mucosa was examined using anti-TLR3 immunohistochemical staining. The expression of TLR3 was observed mainly in the apical part of the supporting cells and in the cytoplasm of acinar cells of Bowman’s glands (Fig. 2a).

To test whether intranasal administration of Poly(I:C) activated the TLR3-mediated signaling pathway in the olfactory mucosa, we performed western blot analysis of phospho-IRF-3 and immunostaining for phospho-NF-κB p65, the downstream signal molecules of TLR3, 8 h after administration of Poly(I:C). Western blot analysis demonstrated that the expression of phospho-IRF-3 was significantly higher in the Poly(I:C) group than in the control group (Fig. 2b; *p* < 0.005). In the control group, anti-phospho-NF-κB p65 immunoreactivity was very weakly observed in the nuclei of a small number of supporting cells (Fig. 2c). On the other hand, in the Poly(I:C) group, intense immunostaining was detected in the nuclei of supporting cells and weak immunostaining was observed in some of the acinar cells of Bowman’s glands (Fig. 2d). These results suggest that Poly(I:C) activated the TLR3-mediated signaling pathway.

### MIP-2 expression level in the olfactory mucosa

**Fig. 3:**
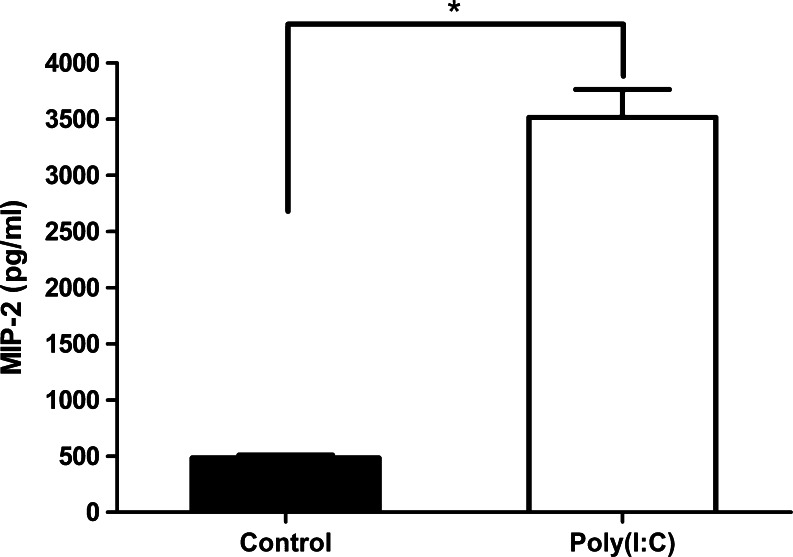
MIP-2 expression in the olfactory mucosa in response to Poly(I:C). MIP-2 concentration in the supernatant of the tissue extracts was measured by ELISA at 12 h after the administration of Poly(I:C) or saline. In the Poly(I:C) group, the MIP-2 level was significantly elevated (*n* = 4, p < 0.001)

We measured the expression level of MIP-2, a neutrophil chemoattractant, in the olfactory mucosa by ELISA. MIP-2 concentration in the supernatant of the tissue extract was 483.1 ± 27.1 pg/ml in the control group and 3517.1 ± 248.9 pg/ml in the Poly(I:C) group. Poly(I:C) induced a significant elevation in the level of MIP-2 (Fig. 3, p < 0.001).

### Morphological changes of the olfactory mucosa by Poly(I:C)

**Fig. 4:**
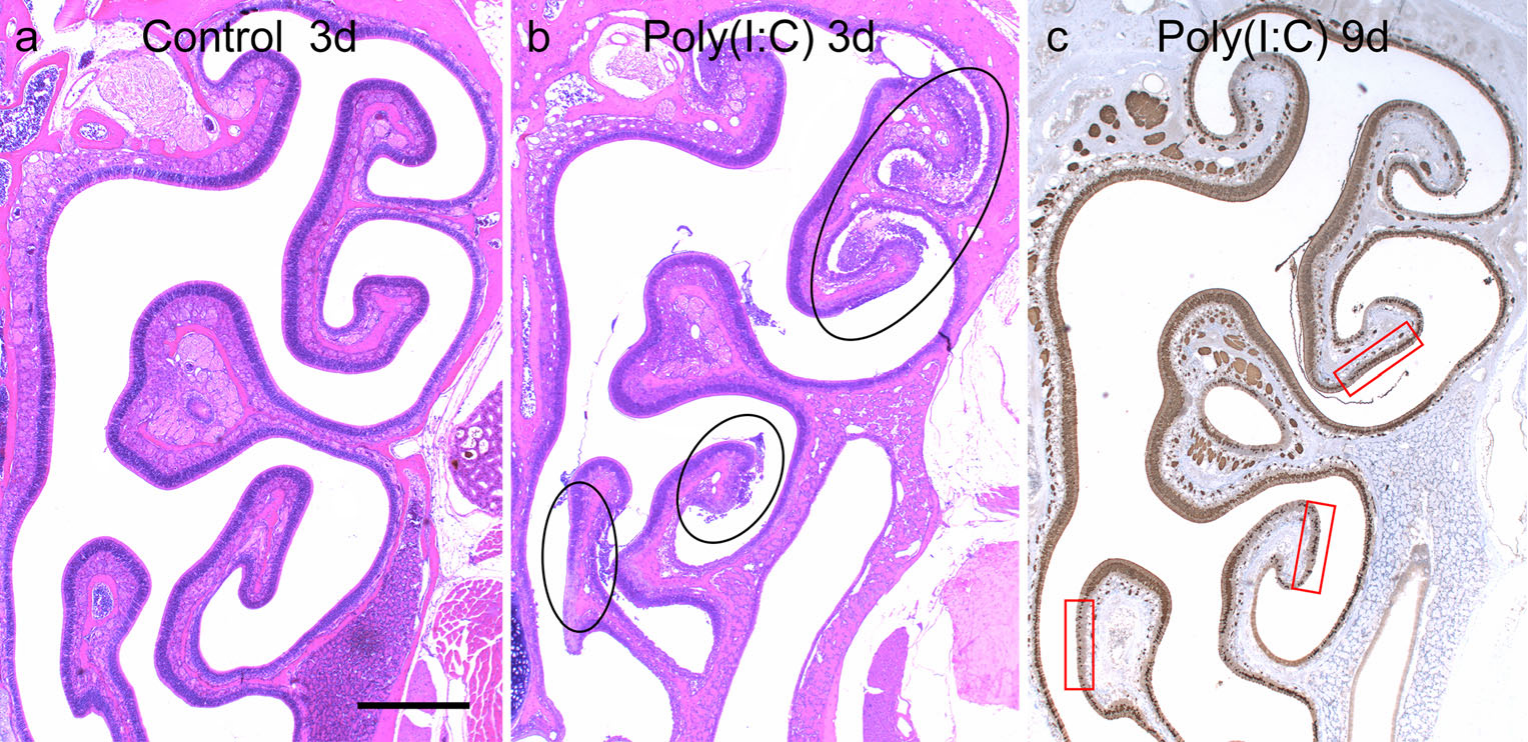
Photomicrographs of the left nasal cavity in the control group (**a**) and the Poly(I:C) group (**b**) at 3 days, and in the Poly(I:C) group (**c**) at 9 days. Sections in (**a**) and (**b**) were stained with H&E, and the section in (**c**) was immunostained with anti-OMP antibody. In the control group, there were no obvious morphological changes (**a**). In the Poly(I:C) group, the olfactory area shows inflammatory and degenerative changes, including the infiltration of inflammatory cells, increasing amount of mucus in the cavity, and the detaching of the neuroepithelium from the basement membrane (**b**). The changes were most severe in the lateral area, including II, IV, and V ethmoturbinates (*black circles* in **b**). At 9 days after the administration of Poly(I:C), the number of OMP-positive ORNs also appeared to have decreased most in the corresponding area (**c**). Three *red rectangles* in (**c**) indicate the microscopic fields along the II, IV and V ethmoturbinates used for histological analyses of neuroepithelial degeneration. *Bar* 0.5 mm

Morphological changes of the olfactory mucosa induced by intranasal administration of Poly(I:C) or saline were examined. At 3 days, in the control group, there were no obvious morphological changes in H&E-stained sections (Fig. 4a). In the Poly(I:C) group, the olfactory area showed inflammatory and degenerative changes, including the infiltration of inflammatory cells, increases in the amount of mucus in the cavity, and detachment of the neuroepithelium from the basement membrane (Fig. 4b).

The degree of the damage was not even throughout the olfactory region: the lateral area, including the II, IV, and V ethmoturbinates, was the most affected (Fig. 4b). This finding was observed in all the mice examined (*n* = 3). Consistent with this finding, at 9 days after the administration of Poly(I:C), the number of OMP-positive ORNs also appeared to have decreased the most in corresponding areas (Fig. 4c). We therefore selected these three areas for histological analyses of neuroepithelial degeneration.

**Fig. 5:**
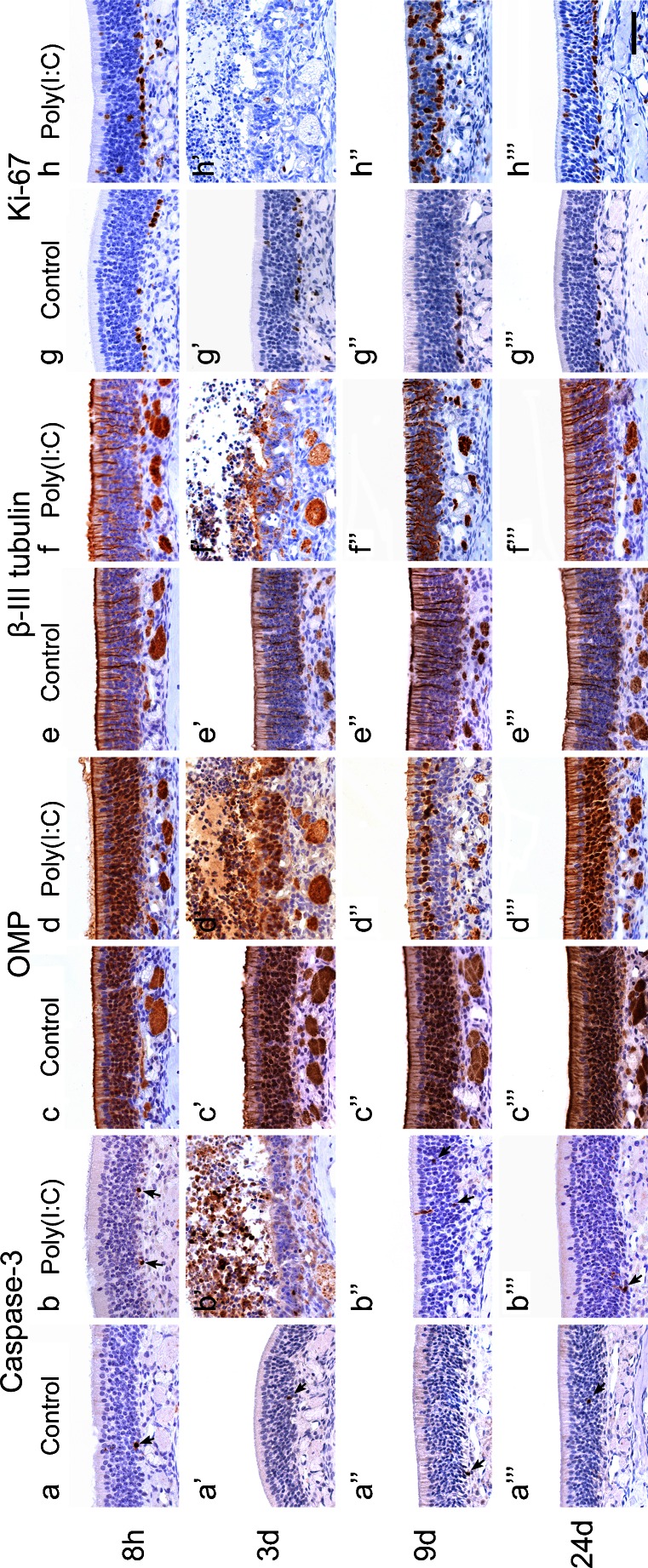
Photomicrographs showing the expression of cleaved caspase-3 (**a**, **b**), OMP (**c**, **d**), βIIIT (**e**, **f**), and Ki67 (**g**, **h**) in the olfactory mucosa of the control group (**a** ,**c**, **e**, **g**) and the Poly(I:C) group (**b**, **d**, **f**, **h**), harvested at 8 h, 3, 9, and 24 days after administration. In the control group, there was no obvious change in the distribution of cells labeled for each antigen throughout the time points (**a**, **c**, **e**, **g**). In the Poly(I:C) group, the number of cleaved caspase-3-positive cells had slightly increased at 8 h (**b**), was robust at 3 days (**b**′), and had returned to control levels by 9 days (**b**′″). In the Poly(I:C) group, a number of OMP-positive ORNs exfoliated and detached from the mucosa at 3 days (**d**′). At 9 days, the number of OMP-positive ORNs had decreased remarkably (**d**″). At 24 days, the olfactory neuroepithelium appeared to have regenerated almost completely (**d**″′). Ki67-positive cells and βIIIT-positive cells had increased at 9 days (**f**″, **h**″) and returned to near control levels at 24 days (**f**″′, **h**″′). *Bar* 50 μm

We next examined the cell dynamics of neuroepithelial cells by using immunohistochemistry for cleaved caspase-3 (apoptotic cells), OMP (mature ORNs), βIIIT (immature ORNs) and Ki67 (proliferating basal cells). In the control group, there were no changes in the distribution of cells labeled for each antigen throughout the time points (Fig. 5a–a″′, c–c″′, e–e″′, g–g″′). In the Poly(I:C) group, the number of apoptotic cells positive for anti-cleaved caspase-3 antibody was slightly increased at 8 h (Fig. 5b), robust at 3 days (Fig. 4b′), and had returned to control levels by 9 days (Fig. 5b″).

**Fig. 6:**
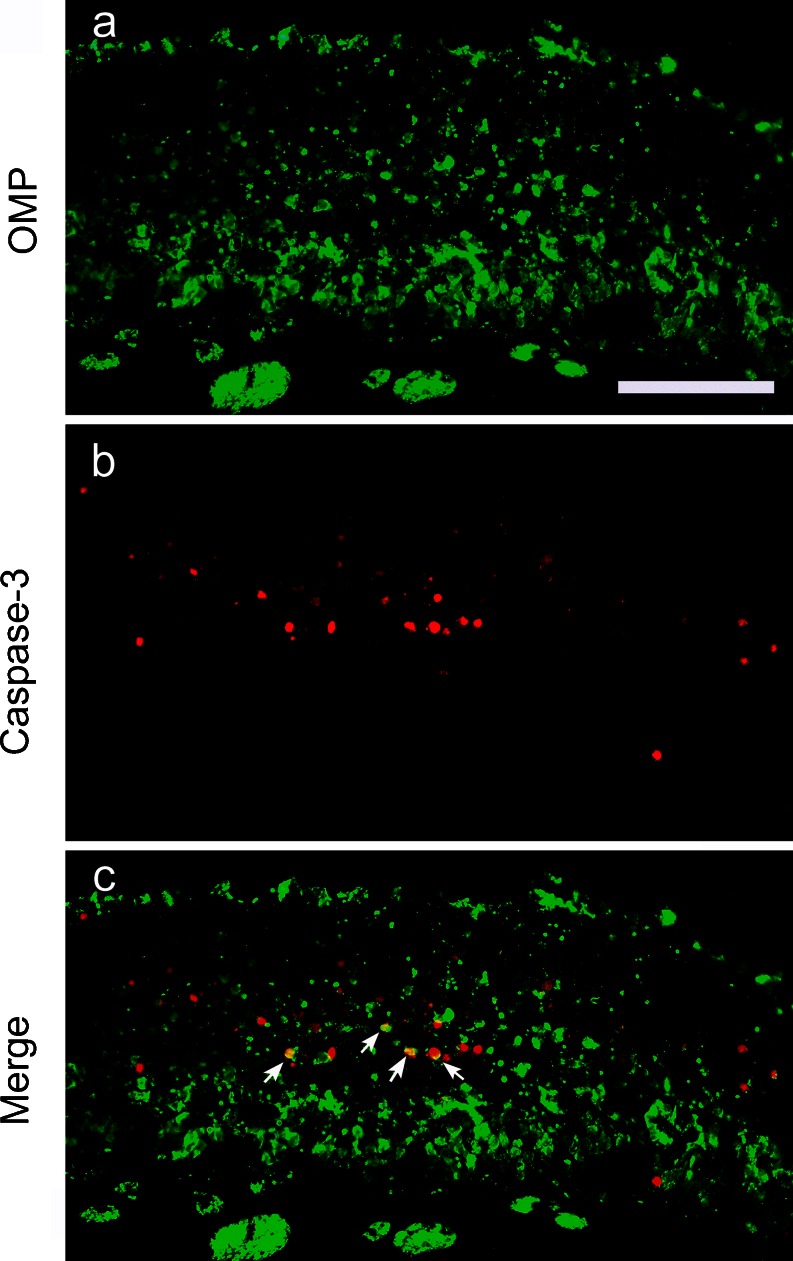
Double-immunofluorescence staining of the olfactory mucosa in the Poly(I:C) group at 3 days with anti-OMP (**a**) and anti-cleaved caspase-3 (**b**) antibodies. The *arrows* indicate cells that are positive for both caspase-3 and OMP. Approximately 3 % of OMP-positive cells were immunopositive for caspase-3, and 30 % of caspase-3 positive cells were immunopositive for OMP.* Bar* 50 μm

In the Poly(I:C) group, OMP-positive ORNs were visually intact at 8 h (Fig. 5d), but by 3 days, they had exfoliated and detached from the mucosa (Fig. 5d′). At 9 days, the number of OMP-positive ORNs had decreased markedly (Fig. 5d″). By 24 days, the olfactory neuroepithelium appeared to have almost completely regenerated (Fig. 5d′″). The result of double-immunofluorescence staining of cleaved caspase-3 and OMP showed that, at 3 days, approximately 3 % of OMP-positive cells were immunopositive for caspase-3, and 30 % of caspase-3 positive cells were immunopositive for OMP (Fig. 6).

In the control group, βIIIT-positive ORNs existed in a single layer above the basal cells (Fig. 5f). Similar to the OMP-positive ORNs, βIIIT-positive ORNs were visually intact 8 h after administration of Poly(I:C) (Fig. 5f′), but they were partially damaged by 3 days (Fig. 5f″). At 9 days, βIIIT-positive ORNs had increased significantly and were present in multiple layers (Fig. 5f″). At 24 days, their numbers had decreased close to control levels (Fig. 5f′″). At 9 days, Ki67-positive cells had also increased and were distributed in all layers of the reconstituting neuroepithelium (Fig. 5h″) and then decreased again by 24 days (Fig. 5h′″).

**Fig. 7:**
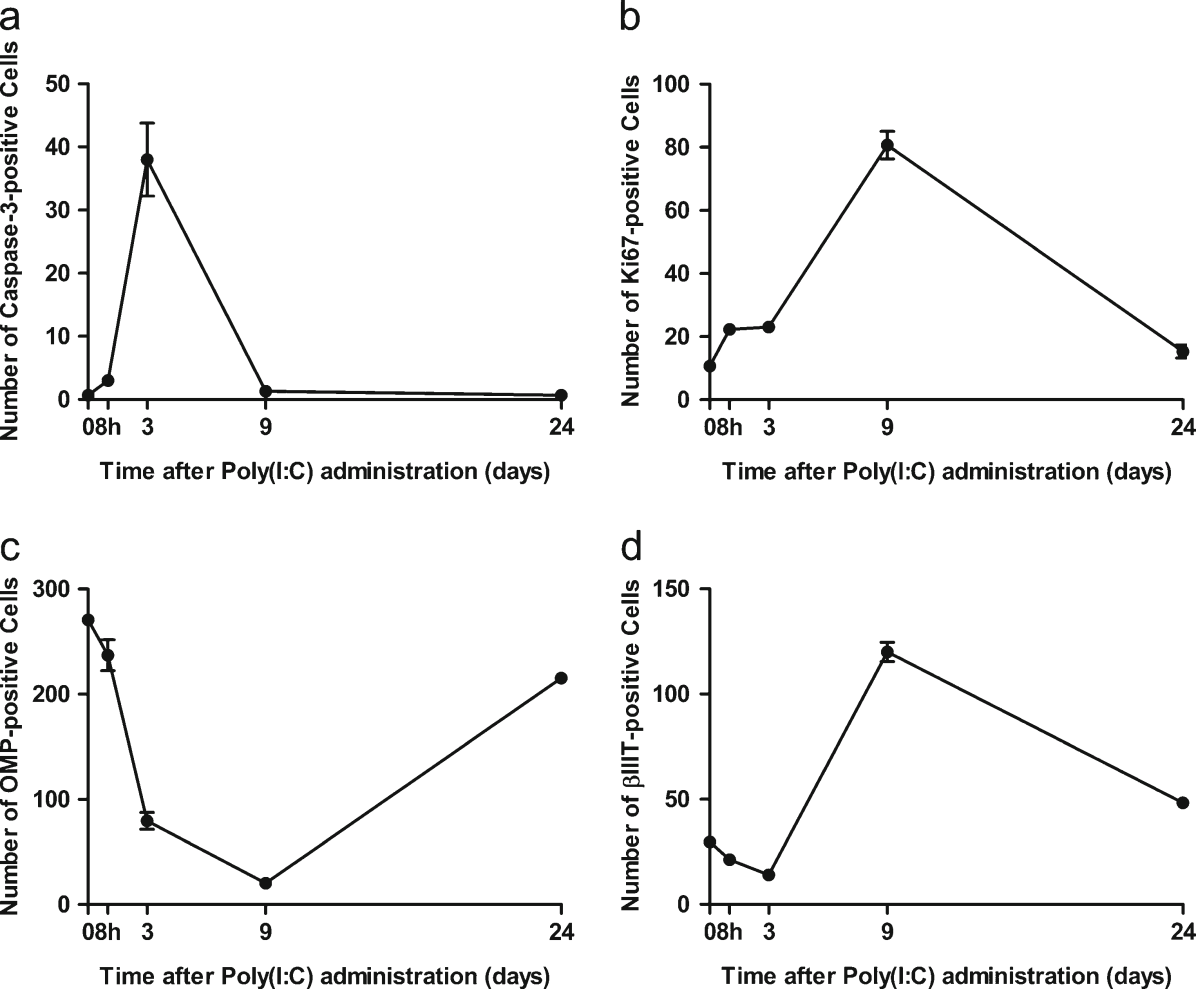
Time course of the number of cells labeled for cleaved caspase-3 (**a**), Ki67 (**b**), OMP (**c**), and βIIIT (**d**) in the olfactory mucosa after intranasal administration of Poly(I:C). Data were collected from three microscopic views (×400) of the II, IV, and V ethmoturbinates, as described in Fig. 3. Three mice were analyzed at each time point

Figure 7 shows the time course of counts of cells labeled for each molecular marker per microscopic field (×400) in the selected areas in the II, IV, and V ethmoturbinates. These quantitative analyses confirmed the quantitative finding described above.

### Poly(I:C)-induced mucus secretion from Bowman’s glands

**Fig. 8:**
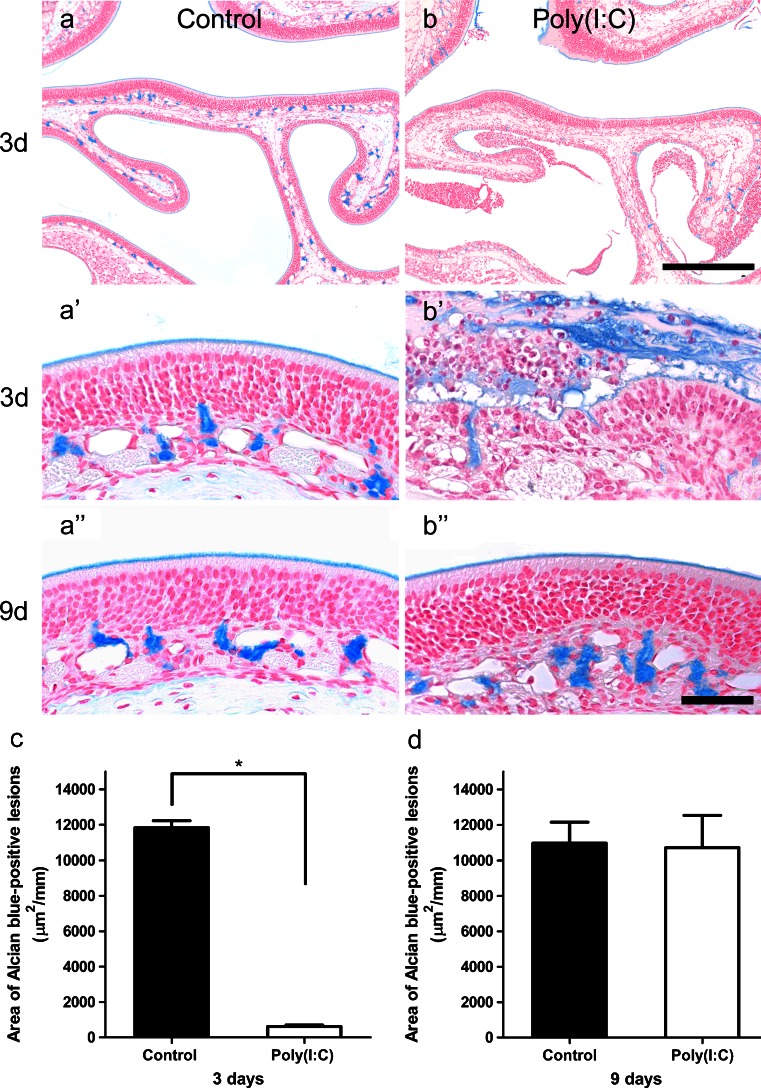
**a**, **b** Photomicrographs of olfactory mucosa of the control group (**a**) and the Poly(I:C) group (**b**) harvested at 3 days (**a**, **a**′, **b**, **b**′) and 9 days (**a**″, **b**″) after the administration of Poly(I:C) or saline, and stained with Alcian blue solution. In the control group, there was no obvious change between 3 and 9 days in the area of Alcian blue-positive mucus in Bowman’s glands (**a**). In the Poly(I:C) group, the mucus in Bowman’s glands had decreased markedly in almost all regions of the left olfactory mucosa at 3 days (**b**, **b**′). At 9 days, the area of Alcian blue-positive mucus in Bowman’s glands appeared to have returned to normal (**b″**).** c**, **d** In quantitative comparisons, the area of Alcian blue-positive mucus at 3 days was significantly smaller in the Poly(I:C) group than in the control group (**c**). At 9 days, there was no significant difference between the two groups (**d**). *Bars* (**a**, **b**) 0.2 mm, (**a**′, **a**″, **b**′, **b**″) 50 μm

In the control group, almost all Bowman’s glands were stained with Alcian blue at 3 days (Fig. 8a) and there was no significant change between 3 and 9 days in the area of Alcian blue positivity (Fig. 8a′, a″). In the Poly(I:C) group, Alcian blue-positive mucus had increased in the nasal cavity and that in Bowman’s glands had decreased markedly in almost all regions of the left olfactory mucosa at 3 days (Fig. 8b, b′). At 9 days, Alcian blue-positive mucus appeared to have returned to normal (Fig. 8b″). The area of Alcian blue-positivity at 3 days was significantly smaller in the Poly(I:C) group than in the control group (Fig. 8c; *p* < 0.0001). At 9 days, there was no significant difference between the two groups (Fig. 8d; *p* = 0.91).

### Behavioral testing for evaluating olfaction

We examined olfactory sensitivity in the mice using an olfactory habituation/dishabituation test before and 9 days after the administration of Poly(I:C), the time at which the number of mature olfactory receptor neurons was the lowest. As the degeneration of olfactory neuroepithelium by Poly(I:C) was most severe in the area corresponding to zone IV in odorant receptor expression (Ressler et al. 1993; Vassar et al. 1993; Nagao et al. 2002), we used propyl propionate as a test odorant because it is considered to be predominantly perceived by zone IV (a database of odor maps from the rat olfactory bulb is available at http://gara.bio.uci.edu/).

**Fig. 9:**
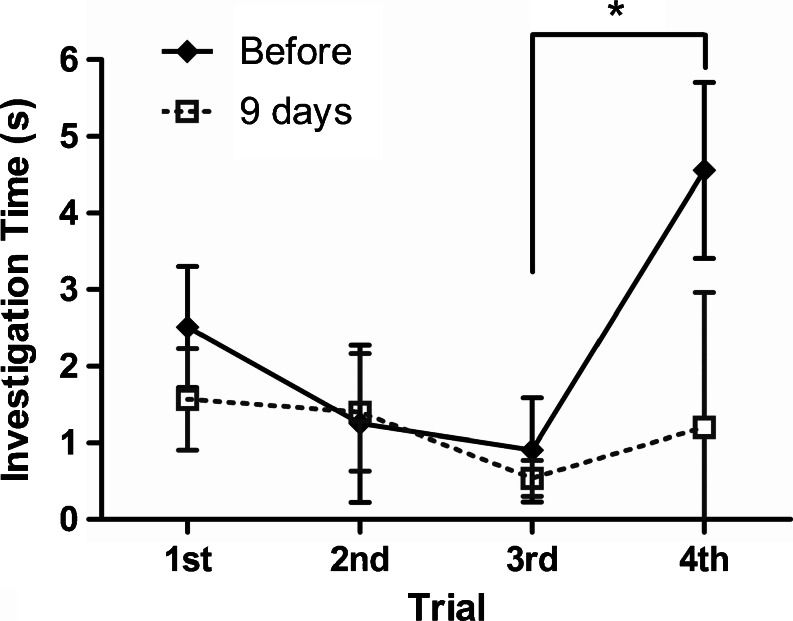
Olfactory habituation/dishabituation test. Mice were presented with a filter paper soaked in mineral oil placed in a polystyrene dish three times at 1 min intervals. In the fourth trial, a filter paper soaked instead with propyl propionate was presented for 3 min.The mouse behavior was recorded with a digital video camera and each investigation time was measured. Before the administration of Poly(I:C), the investigation time on the fourth trial was significantly longer than that on the third trial (*n* = 5, *p* < 0.01). At 9 days after the first administration of Poly(I:C), there was no significant difference between them, suggesting a decrease of olfactory sensitivity (*p* = 0.438)

In the control test, before the administration of Poly(I:C), the investigation times on the second and the third trials decreased step by step due to habituation. However, when the mice were introduced to a new odorant on the fourth dishabituation trial, the investigation time was significantly longer than that on the third trial (*p* < 0.01). The mice which had been administered Poly(I:C) also showed similar decrease in investigation time on the second and the third trials suggesting habituation, but did not show dishabituation on the fourth trial and there was no significant difference in the investigation time between the third and fourth trials (Fig. 9).

### Inflammatory cell infiltration in the olfactory mucosa induced by Poly(I:C)

**Fig. 10:**
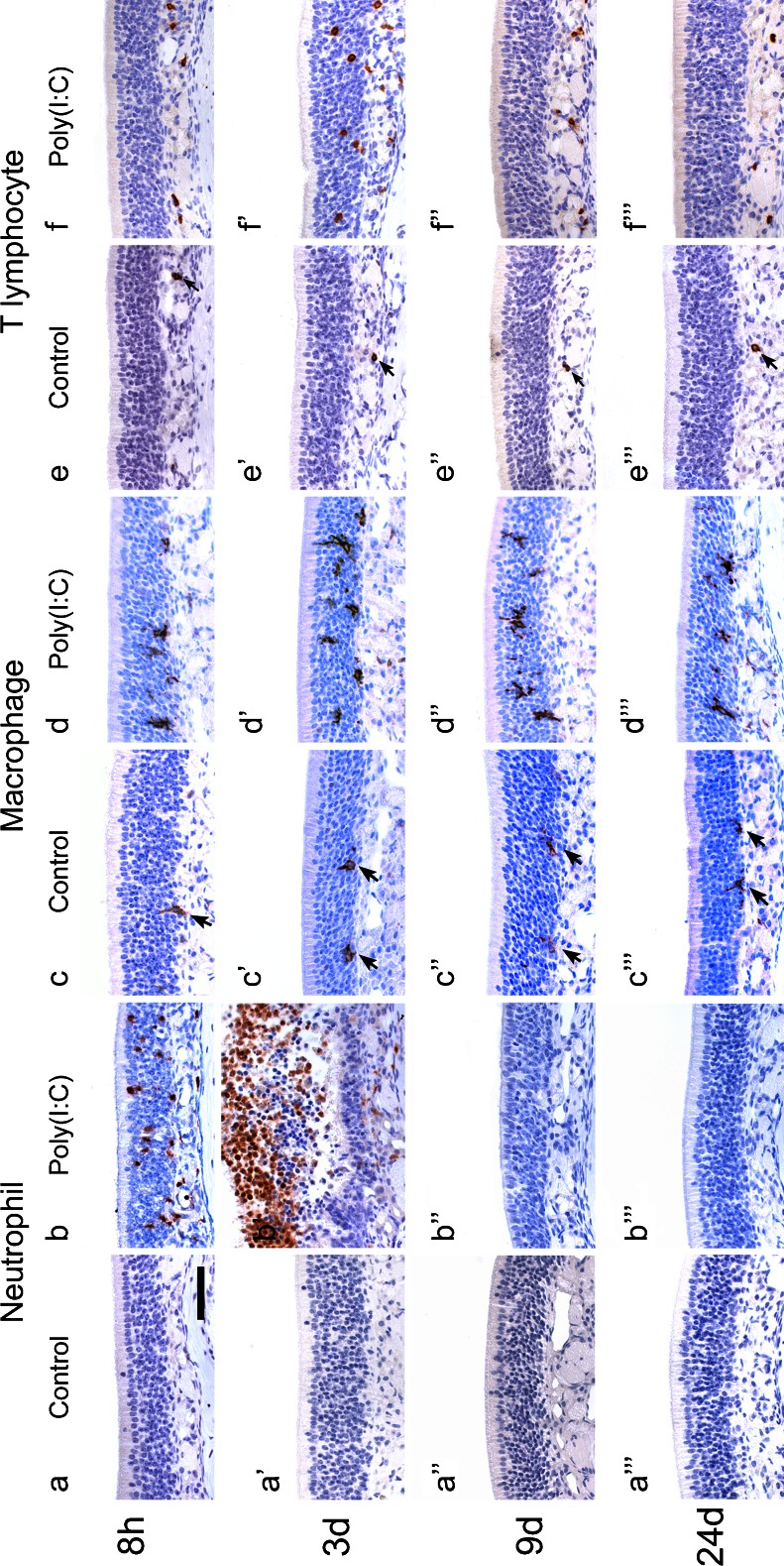
Photomicrographs showing inflammatory cell infiltration in the olfactory mucosa induced by Poly(I:C). Sections of the mucosae harvested at 8 h, 3, 9, and 24 days after the first administration of Poly(I:C) (**b**, **d**, **f**) or saline (**a**, **c**, **e**) were immunostained for neutrophils (**a**, **b**), F4/80 (Macrophage; **c**, **d**) and CD3 (T lymphocyte; **e**, **f**). In the control group, neutrophils were not observed across any of the time points (**a–a**″′). In the Poly(I:C)group, many neutrophils had infiltrated the olfactory neuroepithelium by 8 h (**b**) and exuded into the nasal cavity by 3 days (**b**′). At 9 and 24 days, neutrophils were not observed in the olfactory mucosa (**b**″, **b**″′). In the control group, a small number of macrophages were present in the olfactory neuroepithelium at all the time points (**c**–**c**″′). In the Poly(I:C) group, macrophages were observed mainly in a deep layer of the olfactory neuroepithelium at 8 h (**d**) and in all layers at 3 days (**d**′).At 9 days, their number had slightly reduced (**d**″) but some still remained in the olfactory mucosa at 24 days (**d**″′). In the control group, a small number of T lymphocytes were observed in the lamina propria (**e–e**″′). In the Poly(I:C) group, T lymphocytes were observed mainly in the lamina propria at 8 h (**f**), and by 3 days they had infiltrated the olfactory neuroepithelium (**f**′). At 9 and 24 days, they were observed mainly in the lamina propria (**f**″, **f**″′).* Bar* 50 μm

We examined inflammatory cell infiltration in the olfactory mucosa induced by Poly(I:C) administration. In the control group, neutrophils were not observed in the olfactory mucosa at any of the time points (Fig. 10a–a″′), but after the administration of Poly(I:C), many neutrophils infiltrated the olfactory mucosa (Fig. 10b). They had exuded into the nasal cavity by 3 days (Fig. 10b′). At 9 and 24 days, neutrophils were no longer observed in the mucosa (Fig. 10b″, b″′).

In the control group, a small number of macrophages existed in the olfactory neuroepithelium throughout the time points (Fig. 10c–c″′). At 8 h after the administration of Poly(I:C), macrophages had mainly infiltrated a deep layer of the olfactory neuroepithelium (Fig. 10d). By 3 days, they had infiltrated all layers more robustly (Fig. 10d′), while at 9 days their numbers had slightly reduced (Fig. 10d″). In contrast to neutrophils, macrophages still remained in the olfactory mucosa at 24 days, although their number had decreased significantly compared with that at 3 days (Fig. 10d″′).

In the control group, a small number of T lymphocytes were observed in the lamina propria (Fig. 10e–e″′). At 8 h after the administration of Poly(I:C), T lymphocytes had infiltrated the lamina propria (Fig. 10f) and by 3 days they had infiltrated the olfactory neuroepithelium (Fig. 10f′). After 9 days, they were observed mainly in the lamina propria (Fig. 10f″, f″′).

**Fig. 11:**
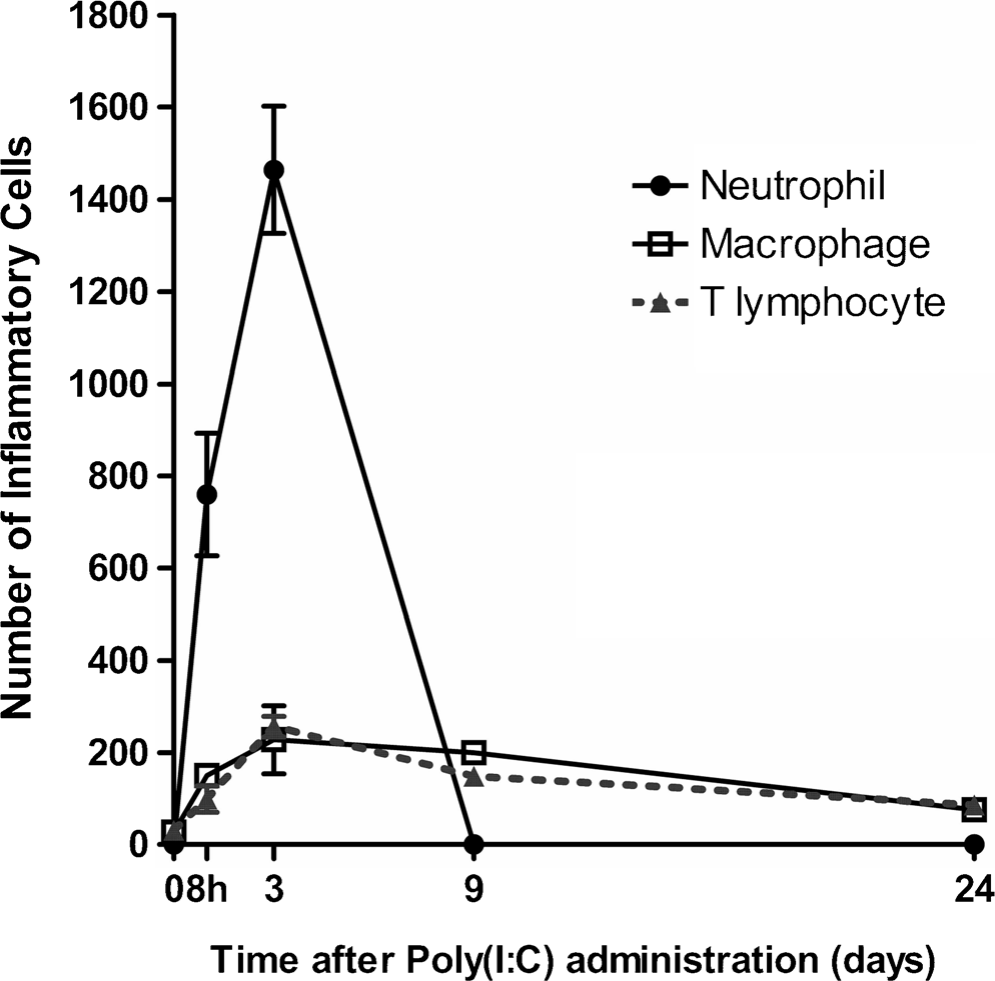
Time course of the number of neutrophils, macrophages, and T lymphocytes in the olfactory area after the administration of Poly(I:C). The total number of each inflammatory cell type in the whole lining of the left olfactory mucosa and in the nasal cavity was counted in three mice at each time point

Figure 11 shows the time course of the total number of each inflammatory cell type in the left olfactory mucosa and nasal cavity, which confirms the qualitative impression described above.

### Degeneration of olfactory neuroepithelium induced by elastase

**Fig. 12:**
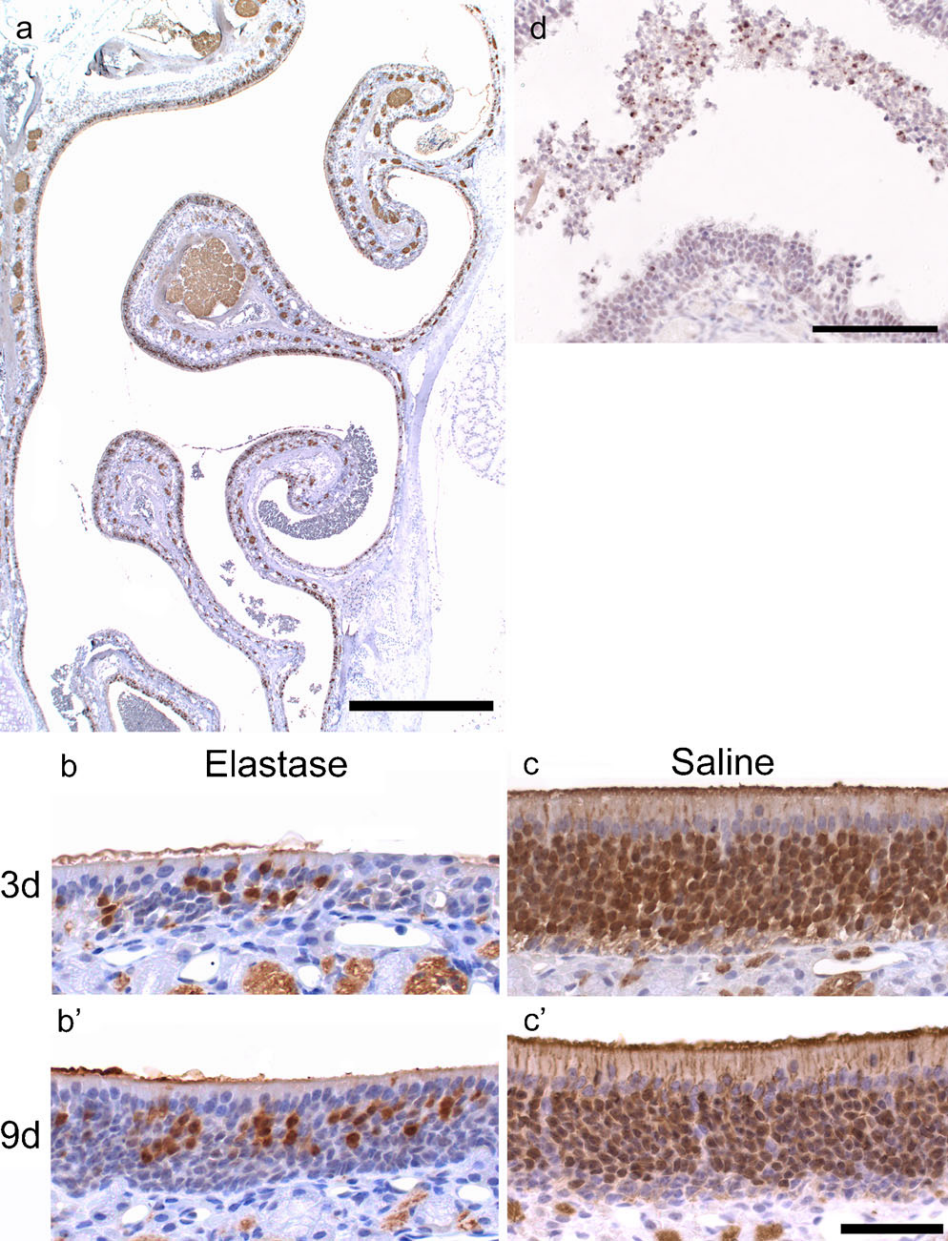
Degeneration of the olfactory neuroepithelium induced by intranasal administration of elastase. Sections of the olfactory mucosa harvested at 3 days (**a**, **b**, **c**) and at 9 days (**b**′, **c**′) after the administration of elastase (**a**, **b**, **b**′) or saline (**c**, **c**′), were immunostained with anti-OMP antibody. At 3 days, the number of OMP-positive ORNs in the neuroepithelium had decreased in almost the whole lining of the olfactory mucosa (**a**). In a high magnification view, the number of OMP-positive ORNs was very low at 3 days after the administration of elastase (**b**). At 9 days, their number still remained reduced, but the neuroepithelial thickness had increased (**b**′). After the administration of saline, there was no change in the olfactory neuroepithelium (**c**, **c**′). Sections of the olfactory mucosa harvested at 8 h after the administration of elastase were immunostained with anti-phospho-NF-κBp65 antibody (**d**). Intense immunoreactivity was detected in the nuclei of exfoliated neuroepithelial cells and weak immunoreactivity was also observed in the nuclei of remaining neuroepithelial cells.* Bars* (**a**) 0.5 mm, (**b**, **c**) 50 μm, (**d**) 100 μm

We examined how the olfactory neuroepithelium could be injured by intranasal administration of elastase. In contrast to Poly(I:C)-treated mice, almost all regions of the olfactory neuroepithelium were homogeneously damaged in elastase-treated mice and the number of OMP-positive ORNs was very low at 3 days (Fig. 12a, b). At 9 days, their number still remained reduced, but the neuroepithelial thickness was increased, probably due to the proliferation of basal cells (Fig. 12b′). There was no obvious morphological change in the olfactory neuroepithelium after the administration of saline (Fig. 12c, c′). We performed immunostaining for phospho-NF-κB p65 on the mucosa at 8 h after administration of elastase. Intense immunoreactivity was detected in the nuclei of exfoliated neuroepithelial cells, but not in as many as observed in the Poly(I:C) group, and weak immunoreactivity was also observed in the nuclei of remaining neuroepithelial cells (Fig. 12d).

### Effects of neutrophil elastase inhibitor on Poly(I:C)-induced neuroepithelial damage

**Fig. 13:**
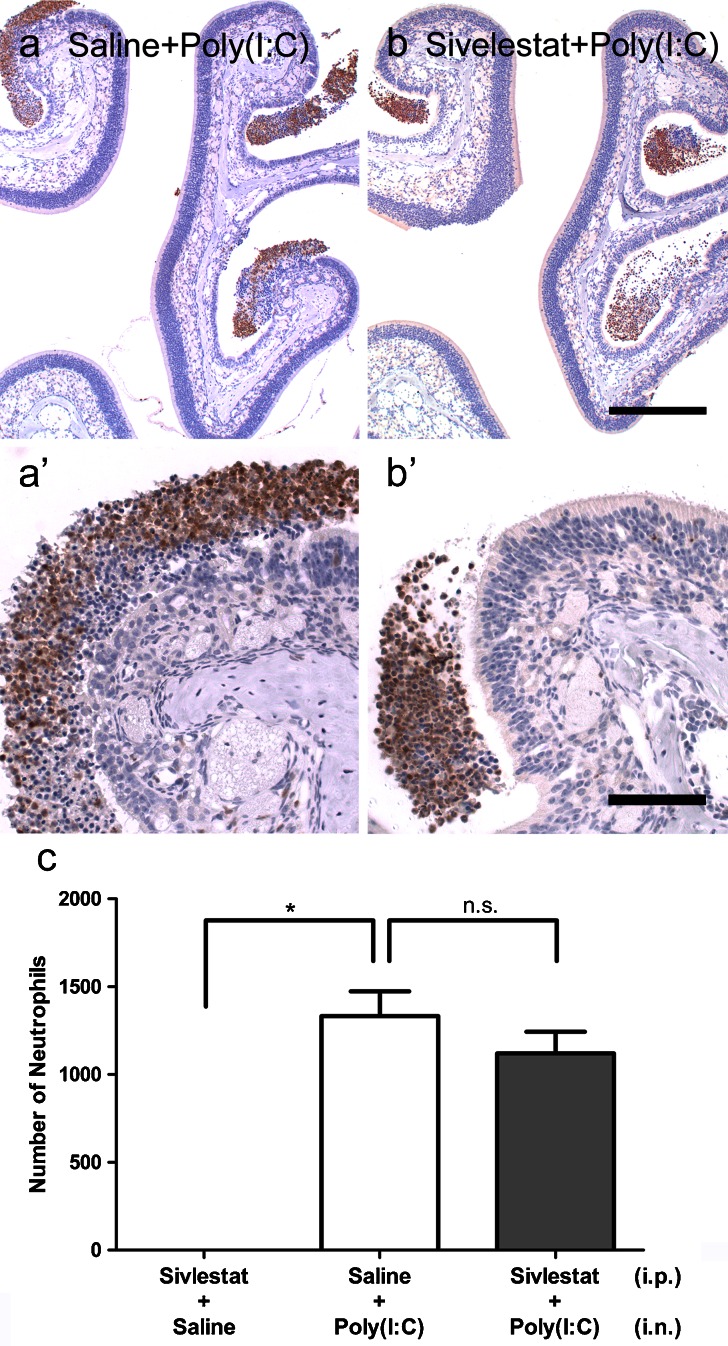
Protective effect of Sivelestat against damage to the olfactory neuroepithelium induced by Poly(I:C). Sivelestat or saline was administered intraperitoneally 30 min before and 12 h after every third intranasal administration of Poly(I:C) or saline. Mice were sacrificed at 3 and 9 days after the first intranasal administration, and sections of the olfactory mucosa were immunostained with anti-neutrophil antibody. **a**, **b** Representative low-magnification (**a**, **b**) and high-magnification (**a**′, **b**′) view photomicrographs of the left nasal cavity in the Saline-Poly(I:C) group and the Sivelestat-Poly(I:C)group, respectively.The total number of neutrophils in the left nasal cavity along the whole mucosal lining and in the nasal cavity was compared between the Saline-Poly(I:C) group and the Sivelestat-Poly(I:C) group. There was no significant difference in the levels of neutrophil infiltration between the two groups (**c**, *n* = 3, *p* = 0.3). *Bars* (**a**, **b**) 0.5 mm, (**a**′, **b**′) 0.2 mm

We examined whether the neutrophil elastase inhibitor, Sivelestat, could suppress the neuroepithelial damage induced by Poly(I:C) and the augmentation of neutrophilic infiltration. Neutrophils were observed in the olfactory mucosa and nasal cavity at 3 days post-administration both in the Saline-Poly(I:C) group (Fig. 13a, a′) and in the Sivelestat-Poly(I:C) group (Fig. 13b, b′). Although the number of neutrophils tended to be lower in the Sivelestat-Poly(I:C) group, there was no significant difference between the two groups (Fig. 13c, *p* = 0.3).

**Fig. 14:**
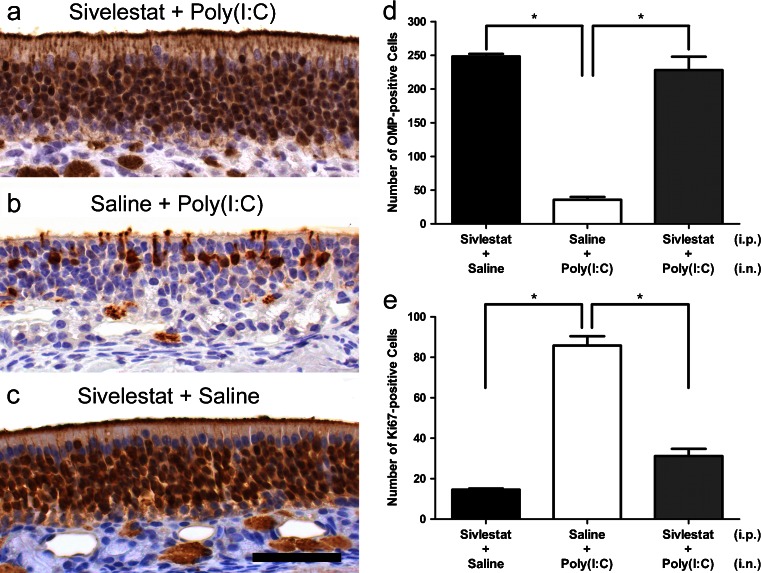
Suppression of the Poly(I:C)-induced neuroepithelial damage by Sivelestat. Mice were sacrificed at 9 days after the administration of Poly(I:C) and the sections of the olfactory mucosa were immunostained with anti-OMP antibody. **a**, **b**, **c** Representative high magnification views of the olfactory mucosa in the Sivelestat-Poly(I:C) group, the Saline-Poly(I:C) group, and the Sivelestat-Saline group, respectively. The number of OMP-positive ORNs was significantly greater in the Sivelestat-Poly(I:C) group than in the Saline-Poly(I:C)group (*p* < 0.0001) (**d**). The number of Ki67-positive cells along the entire length of left olfactory neuroepithelium was significantly lower in the Sivelestat-Poly(I:C) group than the Saline-Poly(I:C) group (*p* < 0.0001) (**e**). *Bar* 50 μm

The neuroepithelial damage in the Sivelestat-Poly(I:C) group (Fig. 14a) was less severe compared with that in the Saline-Poly(I:C) group (Fig. 14b): the number of OMP-positive ORNs was significantly greater in the Sivelestat-Poly(I:C) group than in the Saline-Poly(I:C) group (Fig. 14d; *p* < 0.0001). In contrast, there was no significant difference between the Sivelestat-Saline group and the Sivelestat-Poly(I:C) group (*p* > 0.05). In addition, the number of Ki67-positive cells was significantly smaller in the Sivelestat-Poly(I:C) group than in the Saline-Poly(I:C) group (Fig. 14e, *p* < 0.0001).

### Inhibition of Poly(I:C)-induced neuroepithelial damage in a neutropenic murine model

**Fig. 15:**
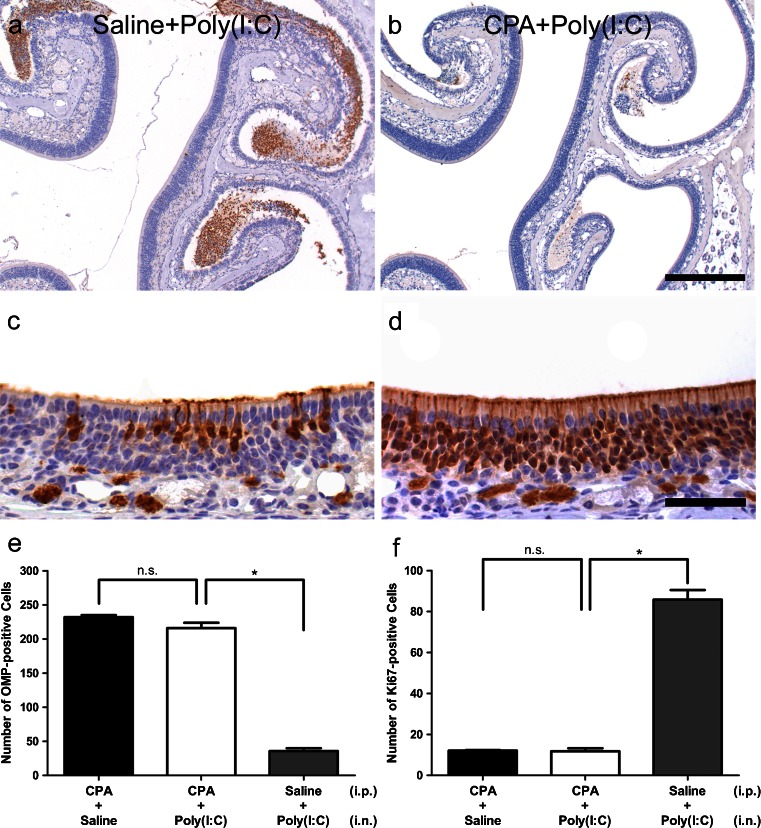
Suppression of neuroepithelial damage in cyclophosphamide (CPA)-induced neutropenic mice. **a**, **b** Representative images of neutrophilic infiltration at 3 days in the Saline-Poly(I:C) group and in the CPA-Poly(I:C) group, respectively. Immunostaining of the olfactory mucosa with anti-OMP antibody in the Saline-Poly(I:C) group and in the CPA-Poly(I:C) group at 9 days are also shown in (**c**) and (**d**), respectively. In quantitative comparisons, the number of OMP-positive ORNs was significantly higher in the CPA-Poly(I:C) group than in the Saline-Poly(I:C) group (*n* = 3, *p* < 0.0001) (**e**). The number of Ki67-positive cells was significantly smaller in the CPA-Poly(I:C) group than in the Saline-Poly(I:C) group (*n* = 3, *p* < 0.0001) (**f**). *Bars* (**a**, **b**) 0.2 mm, (**c**, **d**) 50 μm

We examined whether Poly(I:C)-induced neuroepithelial damage could be suppressed in a neutropenic murine model. First, we examined the morphological appearance of the olfactory mucosa in the mice treated with cycrophosphamide (CPA). Although CPA may be able to inhibit the proliferation of basal cells in the olfactory mucosa and cause morphological changes in the olfactory neuroepithelium, the dose used in this study did not cause any effects on either the olfactory mucosa or epithelium during the observation period (data not shown). We then tested whether the number of infiltrated neutrophils was reduced in neutropenic mice. In the Saline-Poly(I:C) group, a number of neutrophils were observed in the olfactory mucosa and the nasal cavity 3 days after intranasal administration of Poly(I:C) (Fig. 15a). In contrast, in the CPA-Poly(I:C) group, neutrophils were rarely observed (Fig. 15b), although the number of macrophages which had infiltrated the olfactory mucosa appeared to be similar to that in the Poly(I:C) group (data not shown). Degeneration of the olfactory neuroepithelium 9 days after administration of Poly(I:C) in the CPA-Poly(I:C) group (Fig. 15d) was less severe than that in the Saline-Poly(I:C) group (Fig. 15c). The number of OMP-positive ORNs was significantly greater in the CPA-Poly(I:C) group than in the Saline-Poly(I:C) group (*p* < 0.0001; Fig. 15e). Furthermore, the number of Ki67-positive cells was significantly smaller in the CPA-Poly(I:C) group than in the Saline-Poly(I:C) group (Fig. 15f, *p* < 0.0001)

## Discussion

In the present study, we investigated innate immune responses and morphological changes in the olfactory mucosa after intranasal administration of Poly(I:C). We found that (1) a number of neutrophils infiltrated the olfactory mucosa in response to the intranasal administration of Poly(I:C), (2) intranasal administration of elastase severely damaged the olfactory neuroepithelium, (3) a neutrophil elastase inhibitor could suppress the olfactory neuroepithelial damage induced by Poly(I:C), and (4) the olfactory neuroepithelial damage induced by Poly(I:C) was suppressed in the neutropenic murine model. These results suggest that damage to the olfactory neuroepithelium by Poly(I:C) through TLR3 signaling is attributed mainly to the cytotoxic effect of elastase released by neutrophils that infiltrate the olfactory mucosa due to an innate immune reaction. Therefore, such a cellular mechanism is considered to be, at least partly, involved in the pathogenesis of human PVOD.

### Expression of TLR3 in the olfactory mucosa

It has recently been shown that TLR3 is expressed in airway epithelial cells as well as dendritic cells or fibroblasts (Kadowaki et al. 2001; Matsumoto et al. 2002; Gern et al. 2003; Sha et al. 2004; Matsukura et al. 2006). To our knowledge, the present study is the first to demonstrate the expression of TLR3 in the olfactory mucosa. Immunohistochemistry with an anti-TLR3 antibody showed intense labeling in the apical part of supporting cells in the olfactory neuroepithelium. Supporting cells occupy a major part of the surface of the olfactory mucosa and are frequently exposed to foreign substances. Although the function of supporting cells in the olfactory neuroepithelium has not been fully elucidated, previous studies have proposed several possible roles, including mucus production or regulation, scavenging of local odorants, and phagocytosis of apoptotic ORNs (Suzuki et al. 1996; Makino et al. 2009). The results of the current study strongly suggest that the supporting cells also serve as a first line of defense for the immune system by taking foreign materials into the cytoplasm and inducing an immune response.

Although the function of the Bowman’s glands is so far largely unknown, it is speculated that they have functions including the protection of olfactory cilia, transport of odorants (Matarazzo et al. 2002), prevention of mucosal infection through the secretion of antimicrobial agents such as IgA, lactoferrin, and lysozyme (Getchell and Getchell 1991; Mellert et al. 1992), and biochemical detoxification through biotransformation enzymes (Ling et al. 2004). We observed that TLR3 was also expressed in Bowman’s glands. This finding suggests that Bowman’s glands may recognize viruses via TLR3 and act as part of the immune system similar to the supporting cells. This homology between supporting cells and Bowman’s glands appears to fit with their same lineage and similar immunohistochemical profiles (Huard et al. 1998).

In the Poly(I:C) group, Alcian blue-positive mucus in the nasal cavity had increased, while the area of Alcian blue-positivity in Bowman’s glands had decreased at 3 days. The most likely explanation for this finding would be that intranasal administration of Poly(I:C) induced rapid mucus secretion from Bowman’s glands into the nasal cavity and a decrease in the total amount of mucus in Bowman’s glands. Another possibility, not mutually exclusive with the first possibility, is that the toxic effect of Poly(I:C) reduced the production of the mucus in the acinar cells of the glands, and/or the mucus composition in the glands was altered and the Alcian blue-positivity became reduced. Whatever the cellular mechanisms, this histochemical change in Bowman’s glands suggests that they are affected by Poly(I:C) stimulation and possibly contribute to the elimination of pathogens by secreting mucus.

### Poly(I:C)-induced immunological reaction and olfactory neuroepithelial damage

After the administration of Poly(I:C), we observed an increase in the expression of phospho-IRF-3 and phospho-NF-κB p65, downstream signaling molecules of TLR3, and upregulated expression of MIP-2 (mouse homolog of IL-8), a neutrophil chemoattractant, in the olfactory mucosa. Several types of inflammatory cells including neutrophils, macrophages, and T lymphocytes then infiltrated the olfactory mucosa in a time-dependent manner. Neutrophils were the dominant cell type in the early phase of the response. Macrophages and T lymphocytes infiltrated to a lesser extent and remained in the olfactory mucosa relatively longer than neutrophils. It has been reported that, when airway epithelial cells are stimulated by Poly(I:C), they produce leukocyte chemoattractants such as IL-8 and MIP-1α (Matsukura et al. 2006; Berube et al. 2009; Ohkuni et al. 2011), leading to the infiltration of inflammatory cells. IL-8 upregulation following the activation of TLR3 in airway epithelial cells is mediated by the NF-κB and MAPK (mitogen-activated protein kinase) pathways (Berube et al. 2009). In the current study, we observed similar upregulation of TLR3-mediated signaling molecules and MIP-2 in the olfactory mucosa. Therefore, we speculate that the neutrophil influx into the olfactory mucosa was caused by the upregulation of MIP-2 expression, which was induced via the TLR3 signaling pathway activated by Poly(I:C).

Primed neutrophils release several inflammatory mediators into extracellular spaces, and neutrophil elastase in particular can degrade almost all components of the extracellular matrix including elastin, collagen (types I-IV), proteoglycan, fibronectin, and laminin (Ginzberg et al. 2001). Neutrophil elastase also induces the production of cytokines, such as IL-6, IL-8, macrophage inflammatory protein-1(MIP-1), and mucin, from epithelial cells (Sommerhoff et al. 1990; Nakamura et al. 1992; Bedard et al. 1993), resulting in the exacerbation of inflammation. In the present study, the tissue damage was most severe in the lateral area of the olfactory region, where neutrophilic infiltration was marked, suggesting that neutrophils might be involved in the pathogenesis of tissue damage. We also demonstrated that Poly(I:C)-induced olfactory neuroepithelial damage was inhibited by prior administration of the neutrophil elastase inhibitor, Sivelestat. In addition, we demonstrated that the intranasal administration of elastase induced neuroepithelial damage, and that Poly(I:C)-induced neuroepithelial damage was suppressed in the neutropenic murine model. These findings strongly suggest that neutrophil elastase plays an important role in the Poly(I:C)-induced damage of the olfactory neuroepithelium. It has previously been reported that a neutrophil elastase inhibitor reduced the number of neutrophils in a murine model of pneumococcal pneumonia (Yanagihara et al. 2007). In the current study, there was no significant difference in the number of infiltrating neutrophils between the Sivelestat-Poly(I:C) group and Saline-Poly(I:C) group. This result suggests that, although neutrophil elastase is involved in the pathogenesis of the Poly(I:C)-induced neuroepithelial damage, other inflammatory mediators provoke the recruitment of neutrophils in the olfactory mucosa.

Unlike neutrophils, macrophages were observed in the olfactory mucosa long after acute inflammation. Recent studies have shown that macrophages play a central role in the resolution of inflammation and wound repair as well as in the initiation of inflammation (Mantovani et al. 2002; Daley et al. 2010), by phagocytizing apoptotic neutrophils and tissue debris and producing various mediators such as anti-inflammatory cytokines and growth factors. Therefore, macrophages may play a role in the downregulation of inflammation and the repair of olfactory neuroepithelium.

It is important to recognize one limitation of our study. Although we focused on TLR3-mediated cellular and molecular events in the olfactory mucosa, real viral infections can induce other innate immune responses and adaptive immune responses which could also contribute to tissue damage. For example, it has been shown that viral infections can activate a broader set of intracellular innate immune receptors, such as TLR7 or TLR9. By using an experimental model in which a specific molecular pathway is activated, we would be able to demonstrate that TLR3 signaling is one of the components of an immunological reaction during viral infections in the olfactory mucosa. Other signaling pathways could be evaluated using the same methodology.

The result of double-immunofluorescent staining of caspase-3 and OMP revealed that 3 % of cells positive for OMP were also positive for cleaved caspase-3, and that 30 % of cells positive for cleaved caspase-3 were also positive for OMP. These findings suggest that the cell death of ORNs induced by intranasal administration of Poly(I:C) occurred partly due to a caspase-3 mediated apoptotic process, but mostly due to other mechanisms such as necrosis. They also suggest that other cell types in the neuroepithelium, such as supporting cells and basal cells also underwent cell death through apoptotic processes. The pathophysiology of cell death in the olfactory mucosa has been investigated in a number of experimental studies, which have suggested that the mode of cell death varies depending on the etiology. For example, a study using transgenic mice which express TNF-α in supporting cells under the control of doxycycline demonstrated that TNF-α can cause the loss of mature ORNs and infiltration of inflammatory cells into the olfactory mucosa (Lane et al. 2010). In this model, supporting cells were retained despite extensive loss of mature ORNs, suggesting that apoptosis is the primary mode of ORN cell death. In another model, when mice were sensitized to intranasal *Aspergillus fumigatus* extract and subsequently challenged acutely or chronically with the allergen, the mice demonstrated elevated eosinophil infiltration and olfactory sensory neuron apoptosis. However, massive neuronal apoptosis without eosinophil infiltration also occurred in nonsensitized mice after a single dose of the extract (Epstein et al. 2008). This suggests that the fungal allergens could induce ORN apoptosis in both an eosinophilic inflammation-dependent way, and also in a direct toxicity way mediated through TLR2 or 4, the latter of which may be similar to what we observed in our Poly(I:C) models. In a nasosinusitis model infected with *Staphylococcus*, single-strand DNA-, Bcl-2-, or Bax-positive apoptotic cells were detected in olfactory neuroepithelium, and, interestingly, the apoptosis of ORNs in the non-infected (i.e., contralateral) side was also upregulated (Ge et al. 2002). The distribution of apoptotic cells was different between infected side and non-infected side, suggesting that the mechanism underlying ORN apoptosis is different. These findings altogether strongly suggest that there are multiple pathways to induce cell death of ORNs.

It is unclear whether the neutrophil-mediated olfactotoxicity is specific to our Poly(I:C) model. As far as we know, the role of neutrophils in tissue damage has not been investigated in other olfactotoxicity models (Brittebo 1995; Genter et al. 1995; Schwob et al. 1995; Ge et al. 2002; Sakamoto et al. 2007; Epstein et al. 2008; Lane et al. 2010). However, neutrophils generally accumulate in response to tissue damage and release cytotoxic chemical mediators, so neutrophil infiltration may, at least partly, be involved in the exacerbation of tissue damage as a common mechanism of olfactory mucosal injury. It would be interesting to test if the inhibition of neutrophil function used in our study could also reduce tissue damage in other experimental models of olfactory mucosal injury.

Recent experimental studies have reported that intranasal pretreatment with Poly(I:C) limits the severity of subsequent viral infection-induced pathologies, such as herpes simplex virus encephalitis or severe acute respiratory syndrome (Boivin et al. 2008; Zhao et al. 2012). These findings suggest that pretreatment with Poly(I:C) could modulate the subsequent immune responses and suppress virus replication by upregulated expression of interferon and proinflammatory cytokines. Therefore, Poly(I:C), when the dosage and administrating method have been adjusted appropriately, may contribute to host defense against viral infections and serve as a prophylactic agent for viral infections.

### Regeneration of the olfactory neuroepithelium after injury

The olfactory neuroepithelium of the 3-month-old mice used in this study regenerated spontaneously within approximately 1 month by proliferation and differentiation of basal cells. This observation was similar to those made in other experimental models of olfactory neuroepithelial injury (Hurtt et al. 1988; Schwob et al. 1995; Suzukawa et al. 2011). Our experimental model would be useful to investigate the mechanisms of pathogenesis of PVOD and to help develop new therapeutic strategies from the viewpoint of immune reactions. Since the pathogenesis of PVOD may also involve cell death due to the direct action of viral infections on olfactory cells (Schwob et al. 2001; Mori et al. 2002), further study is needed to elucidate the detailed pathogenesis of PVOD, and it is important to select an experimental model appropriate to the purpose of the study.

Viral infections of the upper respiratory tract occur primarily in young and old populations, but only a few cases, usually elderly people, go on to develop PVOD. The reason for this is not clear, but there seem to be several underlying factors: the number of ORNs, regenerative capacity of basal cells, strength of host immune responses, and types of viruses. We would expect considerable variability in the number of ORNs among individuals due to extrinsic and intrinsic factors (Kondo et al. 2009; Holbrook et al. 2011). Elderly people may be more vulnerable to PVOD, due to a subclinical decrease in the number of ORNs and their low regenerative capacity after injury.

## Conclusion

The current study suggests that, in PVOD, the olfactory neuroepithelium is damaged secondarily by innate immune responses and that neutrophils in particular are involved in this pathogenesis. Thus, the inhibition of neutrophil-mediated tissue damage could be a prophylactic strategy for human PVOD.
